# Recent advances in the biosynthesis and industrial biotechnology of Gamma-amino butyric acid

**DOI:** 10.1186/s40643-024-00747-7

**Published:** 2024-03-16

**Authors:** Ripon Baroi Milon, Pengchen Hu, Xueqiong Zhang, Xuechao Hu, Lujing Ren

**Affiliations:** 1 College of Biotechnology and Pharmaceutical Engineering, Nanjing Tech University, No. 30 South Puzhu Road, Nanjing, 211816 People’s Republic of China; 2 Shanghai JanStar Technology Development Co, Ltd., No. 1288, Huateng Road, Shanghai, People’s Republic of China

**Keywords:** Gamma-aminobutyric acid, Biosynthesis, Microbial production, Fermentation optimization, Metabolic pathways

## Abstract

GABA (Gamma-aminobutyric acid), a crucial neurotransmitter in the central nervous system, has gained significant attention in recent years due to its extensive benefits for human health. The review focused on recent advances in the biosynthesis and production of GABA. To begin with, the investigation evaluates GABA-producing strains and metabolic pathways, focusing on microbial sources such as Lactic Acid Bacteria, *Escherichia coli*, and *Corynebacterium glutamicum*. The metabolic pathways of GABA are elaborated upon, including the GABA shunt and critical enzymes involved in its synthesis. Next, strategies to enhance microbial GABA production are discussed, including optimization of fermentation factors, different fermentation methods such as co-culture strategy and two-step fermentation, and modification of the GABA metabolic pathway. The review also explores methods for determining glutamate (Glu) and GABA levels, emphasizing the importance of accurate quantification. Furthermore, a comprehensive market analysis and prospects are provided, highlighting current trends, potential applications, and challenges in the GABA industry. Overall, this review serves as a valuable resource for researchers and industrialists working on GABA advancements, focusing on its efficient synthesis processes and various applications, and providing novel ideas and approaches to improve GABA yield and quality.

**Graphical Abstract:**

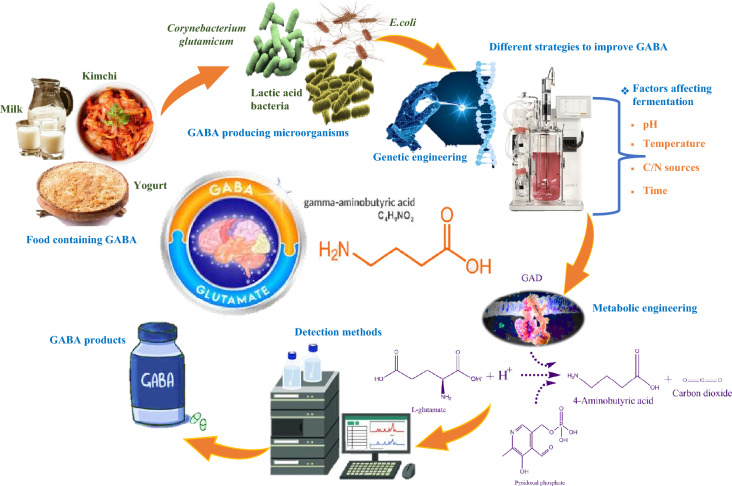

**Supplementary Information:**

The online version contains supplementary material available at 10.1186/s40643-024-00747-7.

## Introduction

GABA is a non-protein amino acid with a four-carbon chemical formula of C_4_H_9_NO_2_ (Dahiya et al. 2021) and a molecular weight of 103.12. GABA is the primary inhibitory neurotransmitter in the mammalian central nervous system and participates in the regulation of many life processes. As a result, it can lower blood pressure, treat anxiety and arrhythmia, control hormone secretion, and enhance liver and kidney functions, among other medical and health benefits. The Chinese Ministry of Health authorized GABA as a new food resource in 2009 (Heli et al. 2022), enabling it to be added to drinks, cocoa goods, and chocolate with a cap on the quantity applied. Similar approvals have already been given in Japan, Europe, and the US, which has given GABA a wide range of application possibilities in the sectors of chemical engineering, food, and medicine. The widespread application of GABA is attributed to the gradual revelation of its physiological functions. GABA and its receptors have also been found in the peripheral nervous system, the endocrine system, and other non-neural organs where it is involved in oxidative metabolism. It is a powerful pain reliever, modulates cardiovascular function, and is used in the treatment of strokes. GABA has been shown to be useful in the treatment of a variety of neurological illnesses, including Parkinson’s disease, Huntington’s chorea (Danduga et al. 2018), and Alzheimer’s disease. It can also raise plasma concentrations, growth hormone levels, and protein synthesis in the brain (Cho et al. 2007). Furthermore, GABA promotes cancer cell death and inhibits cancer cell growth. It is utilized as a bioactive component in the food and medication industry (Kim et al. 2009).

Biosynthesis, chemical synthesis, and plant enrichment all allow the production of GABA. In chemical synthesis, GABA has high purity, but the production cost is high with poor safety and a polluted environment, and the reaction conditions are difficult to control. In addition, GABA obtained by chemical synthesis cannot be incorporated into food, nor can it be consumed as a natural food additive. Moreover, plant enrichment improves the nutritional value of raw materials and is eco-friendly. It, however, has a small size and is difficult to purify. As far as biosynthesis is concerned, it is divided into two categories: direct fermentation and biotransformation. GABA is created via an enzymatic process or a spontaneous microbial fermentation. The microorganisms utilized in the biosynthetic process include lactic acid bacteria (LAB), yeast, *Escherichia coli*, and Aspergillus (Luo et al. 2021; Wang et al. 2018a). LAB, which are generally recognized as safe (GRAS) microorganisms in terms of food safety, have been found to exhibit a higher capacity for fermented GABA production compared to other microorganisms (De Filippis et al. 2020). Glutamic acid decarboxylase (GAD) enables the biosynthesis of GABA via the enzymatic transformation of l-glutamic acid, employing pyridoxal-5′-phosphate (PLP) as an indispensable coenzyme (Yogeswara et al. 2020b). Among all the production methods of GABA, the fermentation of LAB has been acknowledged as a secure and environmentally friendly approach (Evivie et al. 2017). Currently, there are GABA-enriched functional foods available on the market, including cereals, dairy products, and Chinese tea. The health-promoting properties of GABA in meals produced with LAB have been a subject of research interest, leading to a focus on GABA synthesis through the utilization of LAB. The global market for GABA is being propelled by its growing utilization in various sectors such as pharmaceuticals, healthcare, food and beverage, animal feed, and others. In recent years, numerous researchers have conducted comprehensive investigations into the structure, function, and significance of the GABA molecule across various organisms, including plants, animals, and microbes.

**Fig. 1:**
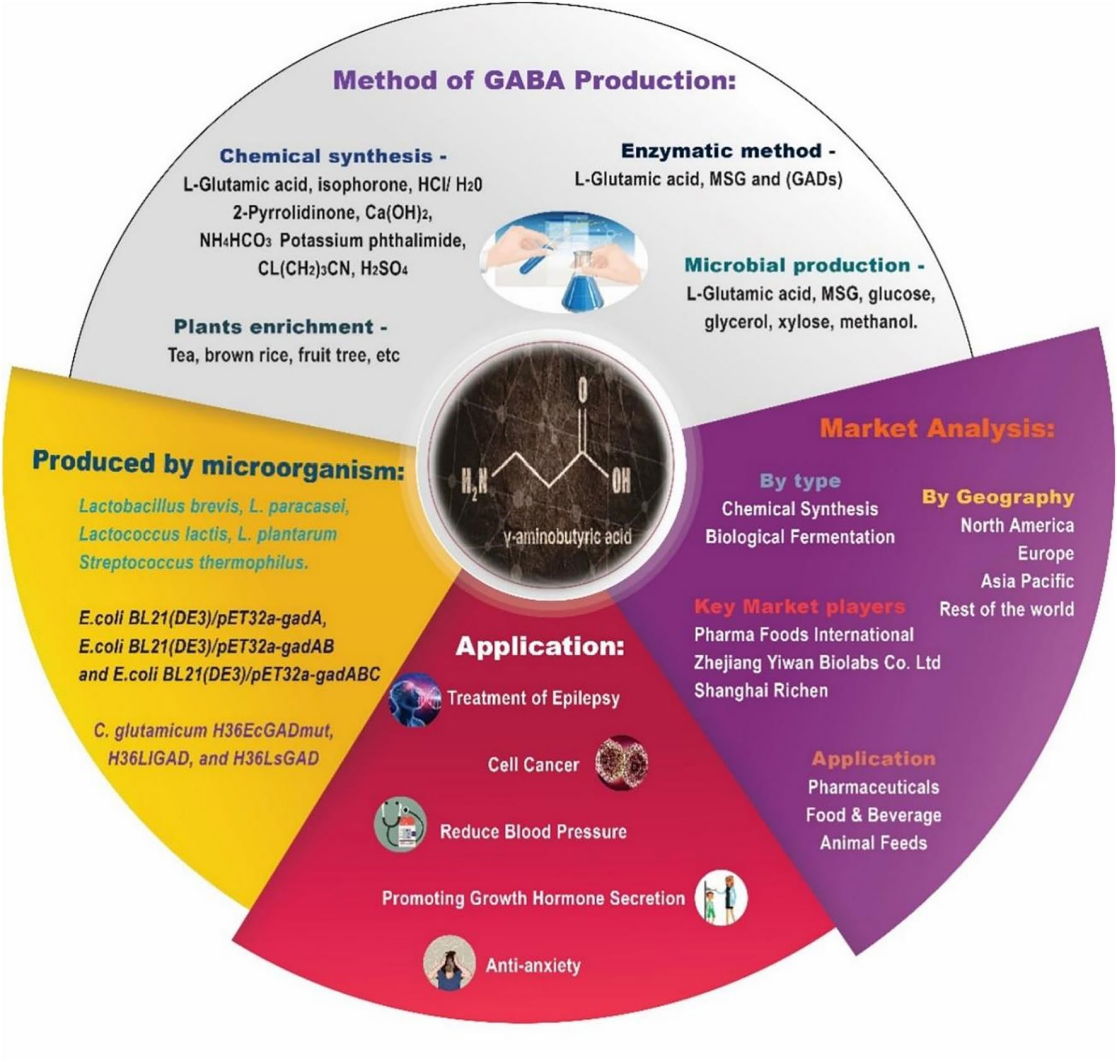
Summary of this GABA review

This comprehensive review article aims to offer valuable insights into various aspects of GABA manufacturing techniques, market dynamics, and development prospects. By synthesizing and analyzing existing literature and research findings (Fig. 1), we delve into the production and functional aspects of GABA, as well as strategies to enhance GABA production. These strategies include co-culture approaches, two-step fermentation methods, diverse techniques for assessing GABA productivity, and discussions on modified metabolic pathways related to GABA synthesis. Furthermore, we offer a concise overview of recent market trends relevant to GABA. Our objective is to furnish researchers, producers, and industry professionals with a broad understanding of contemporary GABA production methods and market dynamics, empowering them to make well-informed decisions and fostering innovation in this burgeoning field.

## Importance of GABA in food

The growing interest in GABA-enriched foods has led to a comprehensive understanding of their biological and health benefits. GABA-enriched foods can be broadly classified into various categories, including cereals, vegetables, fruits, beverages, dairy products, and others. As shown in (Table 1), the content of GABA in each food variety ranges. While natural food sources of GABA are abundant, the amounts present are generally low. To address this limitation, the food technology sector has explored various methods to enhance GABA content in foods, including chemical synthesis and microbial fermentation. However, chemical synthesis raises concerns due to the use of toxic reagents, while microbial fermentation faces challenges related to the purification process.

**Table 1:** The content of GABA in each food variety ranges

Food category	Examples	Content of GABA	References
Cereals	Brown rice (*Oryza sativa* L.)	5.28–27.00 mg/100 g	Munarko et al. (2021)
	Red rice (*Oryza sativa* L.)	1.18–2.91 mg/100 g	Müller et al. (2021)
	Wheat (*Triticum aesticum* L.)	4.55–14.68 mg/100 g	Zhao et al. (2023), Ding et al. (2018)
	Corn (*Zea mays* L.)	15.27 mg/100 g	Paucar-Menacho et al. (2017)
	Barley (*Hordeum vulgare* L.)	1.96–54.00 mg/100 g	Rico et al. (2020)
Vegetables	Tomato (*Solanum lycopersicum* L.)	219.86–404.89 mg/100 g	Suhel et al. (2023)
	Spinach (*Spinacia oleracea* L.)	232.10–381.00 mg/100 g	Pencheva et al. (2022)
	Potato (*Solanum tuberosum*)	44.86 mg/100 g FW	Pencheva et al. (2022)
	Eggplant (*Solanum melongena* L.)	23.28–38.12 mg/100 g	Suhel et al. (2023)
	Parsley (*Petroselinum crispum*)	28.18 mg/100 g FW	Pencheva et al. (2022)
	Beetroot (*Beta vulgaris* subsp.* Vulgaris*)	18.84 mg/100 g FW	Pencheva et al. (2022)
Fruits	Grape (*Vitis vinifera* L*.*)	58.93–109.83 mg/L FW	Gutiérrez-Gamboa et al. (2018)
	European gooseberry (*Phyllanthus emblica*)	10.95 mg/100 g FW	Pencheva et al. (2022)
	Kiwifruit (*Actinidia chinensis*)	2.54–19.14 mg/100 g	Choi et al. (2022)
	Strawberry (*Fragaria* × *ananassa*)	1.5 to 3.5 mg/100 g	Pencheva et al. (2022)
	Apple (*Malus domestica*)	10.0 mg/100 g FW	Pencheva et al. (2022)
Legumes	Red Lentils (*Lens culinaris*)	68.54 mg/100 g DW)	Pencheva et al. (2022)
	Black soybean (*Glycine max* (L.))	4.38–61.00 mg/100 g	Vann et al. (2020)
	Chickpea (*Cicer arietinum* L.)	6.42 mg/100 g	Ferreira et al. (2019)
	Groundnut (*Arachis hypogaea* Linn)	0.56 mg/100 g	Hung and Chen (2022)
Pseudo-cereals	Quinoa (*Chenopodium quinoa* Willd.)	7.00–66.10 mg/100 g	Zhang et al. (2021)
	Tartary buckwheat (*Fagopyrum tataricum* (L.) Gaertn)	1.20 mg/100 g	Peng et al. (2017)
Beverages	White tea (*Camellia sinensis*)	3.49–207.00 mg/100 g	Yılmaz et al. (2020)
	Green tea (*Camellia sinensis*)	0.24–87.00 mg/100 g	Yılmaz et al. (2020)
	Black raspberry juice (*Rubus occidentalis*)	2.76 mg/100 g	Kim et al. (2009)
	Fermented Mulberry Juice (*Morus nigra*)	3.31 mg/mL	Kanklai et al. (2020)
Dairy products	Yogurt	29.96 mg/100 g	Hussin et al. (2020)
	Cheese with *L. lactis* spp. *lactis* as starter	3.20 mg/100 g	Pouliot-Mathieu et al. (2013)
	Cheese with *Kluyveromyces marxianus* B13-5	4.89 mg/100 g	Li et al. (2022a)
Others	Fermented sausage	1.74–2.51 mg/100 g	Yu et al. (2017)
	Chocolate	21.09 mg/100 g	Koh et al. (2023)
	Oyster mushroom (*Pleurotus pulmonarius*)	32.15–57.73 mg/100 g	Wang et al. (2022)
	Shiitake mushroom (*Lentinula edodes*)	17.00–35.00 mg/100 g	Chen et al. (2015)

To overcome these issues, researchers have investigated alternative approaches to increasing GABA content in foods. According to Oh et al. (2019), the use of anaerobic treatment is the investigation’s most important development. This technique entails establishing an oxygen-free atmosphere to promote the synthesis of GABA by certain bacteria via the glutamate decarboxylation process. Under these conditions, it capitalizes on the increased activity of glutamate decarboxylase (GAD). Furthermore, Yu et al.’s (2019a) work explores the application of microorganisms, namely lactic acid bacteria, in the microbial fermentation process to produce GABA spontaneously. This is the process by which food products transform glutamate into GABA. Sun et al. (2022) talked about enrichment technologies that include adding GABA directly to food or creating environments that encourage its production. This can be accomplished through the use of advanced breeding methods or the introduction of microorganisms that produce GABA. Apart from this finding, Ji et al. (2020) looked at the application of salt treatment to regulate osmotic pressure in food environments. Through the activation of certain enzymes, especially those found in fermented foods or by microorganisms themselves, this therapy promotes the production of GABA. The research by Li et al. (2018) suggests that abrupt temperature fluctuations might trigger cellular stress responses, which in turn cause a natural increase in GABA synthesis. The study by Waleed et al. (2020) claims that during the sprouting process, germination causes the activation of several enzymes, including GAD. GABA levels rise as a result of grains and legumes. According to Jiao and Gu’s research from 2019, UV light exposure causes stress reactions in microorganisms and plants, which in turn stimulates the production of GABA. Additional research by Xia et al. (2019) shows that food is altered physically and biochemically by high-pressure processing, which in turn activates enzymes like GAD to increase the synthesis of GABA. The study carried out by Ji et al. (2021) examines the application of sonic waves to induce cell wall breakdown by ultrasound technology, leading to an increase in GABA production. The research conducted by Zargarchi and Saremnezhad (2019) shows that cold plasma treatment causes reactive species to develop in the meal, which modifies the action of enzymes to increase GABA synthesis. Chen et al. (2016) increased the release and activity of the enzymes responsible for GABA production by using pulsed electric field technology to increase the permeability of cell membranes. These diverse and creative methods, each with a unique method of operation, represent important developments in the world of food technology. In response to the increased consumer demand for functional meals that offer health benefits beyond basic nutrition, advancements in food science are being made with the goal of improving food products’ nutritional value. This advancement in food technology demonstrates a commitment to deepening our understanding of food science and how it may be applied to enhance human health and wellbeing.

### Function and application of GABA

GABA has an influence on cognitive functions including cognition, emotion, and memory and is crucial for controlling central nervous system activity. It inhibits nerve transmission, hence lowering neural excitability. By preventing nerve transmission, GABAergic neurons, which are present in the brainstem, basal ganglia, hypothalamus, thalamus, and hippocampus, influence neuronal excitability. GABA, which is present in between 30 and 40% of neurons, is released into synapses to slow the propagation of action potentials (DNA Learning Center 2020). In 1950, Florzy’s team discovered GABA in animals of high taxonomy, revealing its function in nerve impulse conduction. It transmits nearly half of the information regarding feedback inhibition. Krnjevic and colleagues discovered that GABA transmits signals and conveys inhibitory feedback, thereby potentially regulating physiological processes such as human perception and activity (Connor et al. 2010; Jiang et al. 2013; Xu et al. 2017). Therefore, the GABA function’s capacity to regulate neuronal activity and encourage relaxation emphasizes how crucial it is for maintaining a healthy, functional neurological system.

### Treatment of epilepsy

Epilepsy, a chronic neurological disorder characterized primarily by seizures, affects over 50 million individuals globally and presents a significant public health challenge (Bastide et al. 2016). The pathophysiology of epilepsy is complex, involving multiple factors strongly linked to the brain’s central nervous system, with GABA concentrations playing a crucial role. The seminal discovery by Tower et al. in 1961, indicating that GABA in the brain could prevent the onset of epilepsy, marked a pivotal moment in understanding the disorder’s neurochemical underpinnings (Okada et al. 2000).

Current research suggests that the regulation of epilepsy by the central nervous system is intricately tied to changes in ion transport efficiency, synaptic connections, and the activity of neurotransmitters such as glutamate and GABA (Dahlin and Prast-Nielsen 2019; Iimure et al. 2009). Notably, DeLorey and Olsen’s study on the disruption of pyridoxal phosphate (PLP) metabolism in rats, which led to decreased GABA levels and spontaneous seizures, further underscores the critical relationship between epileptogenesis and alterations in the GABAergic network (DeLorey and Olsen 1999). Moreover, lower levels of GABA in brain tissues have been associated with more severe forms of epilepsy and psychosis, underscoring the broader impact of GABA dysregulation (Makino et al. 2008; Savage et al. 2018).

The modulation of GABA availability and activity is a key strategy in epilepsy treatment. Anticonvulsant drugs such as felbamate, valproate, and gabapentin are designed to regulate GABA concentrations, often replacing traditional medications like benzodiazepines and phenobarbital due to the latter’s propensity for side effects and drug resistance (Abou-Khalil 2019). Additionally, emerging evidence links epilepsy with alterations in the gut-brain axis, specifically dysbiosis. Probiotic supplementation has been shown to not only reduce seizure frequency but also enhance GABA activity and improve oxidative balance, offering a novel therapeutic avenue (Bagheri et al. 2019). So, the management and understanding of epilepsy necessitate a multifaceted approach, considering both neurochemical imbalances, particularly in GABAergic signaling, and broader physiological interactions, such as those within the gut-brain axis. This integrated perspective is crucial for developing more effective and comprehensive treatment strategies for this complex and impactful neurological disorder.

### Reduce blood pressure

GABA plays a crucial role in addressing cardiovascular health issues such as arteriosclerosis, hypertension, and increased blood viscosity, especially prevalent in the elderly demographic, which are major risk factors for severe conditions including cerebral hemorrhage, coronary artery disease, myocardial infarction, and stroke. The effectiveness of GABA in these areas has been illuminated through a variety of animal and clinical studies. For instance, GABA’s ability to reduce arterial stiffness and blood viscosity has been noted as a significant factor in mitigating hypertension and cardiovascular risks, as it improves blood vessel elasticity and facilitates smoother blood flow, thereby reducing cardiac workload (Ma et al. 2015). Furthermore, GABA’s role as a neurotransmitter in the central nervous system, where it exerts a post-synaptic inhibitory effect, particularly in the autonomic nervous system, is essential in modulating blood pressure. This is complemented by its action on the vasomotor center of the spinal cord, leading to vasodilation and a consequent decrease in blood pressure (Jewett and Sharma 2018). Recent research has indicated that GABA’s effects may transcend the brain’s boundaries. Protein kinase B (Akt)/glycogen synthase kinase-3 (GSK-3) signaling is inhibited via a β-arrestin-dependent pathway when GABA receptors are activated. The expected impact of this protein kinase activity inhibition is vasodilation, which in turn leads to a reduction in blood pressure. GABA promotes vasodilation and inhibits sympathetic nerve activity, thereby contributing to blood pressure regulation (Heli et al. 2022; Lu et al. 2012). Additionally, a crucial component of the renin-angiotensin system (RAS), which controls blood pressure and fluid homeostasis, is angiotensin-converting enzyme (ACE). By acting as a natural ACE inhibitor, GABA decreases ACE activity and increases vasodilation. Vasoconstriction is counterbalanced by this decrease in angiotensin II levels, which reduces vascular resistance and preserves healthy blood pressure. GABA plays a significant role in cardiovascular health as well as neurological functions, contributing to the maintenance of optimal blood pressure and serving as a potential therapeutic agent in the management of hypertension and other cardiovascular disorders. It plays a crucial inhibitory function within the central nervous system (Patten et al. 2016). Collectively, these diverse mechanisms highlight the significant impact of GABA on cardiovascular health, particularly in addressing the complex challenges associated with cardiovascular conditions in aging populations (Mills 2021).

### Anti-anxiety

Globally, mental illnesses such as anxiety and melancholy have increased over the past few decades, influencing daily life. These chronic or episodic disorders can result in despondency, apathy, remorse, difficulty sleeping, fatigue, and inattention. Anxiety disorders, such as generalized anxiety disorder, panic disorder, phobias, social anxiety disorder, obsessive–compulsive disorder (OCD), and post-traumatic stress disorder (PTSD), are marked by anxiety and fear and can range in severity from moderate to severe (World Health Organization 2017). Depression and anxiety may arise as a consequence of physiological disruptions, with scientific investigations mostly centered on alterations in monoamine production. Recent research has shown neuro-endocrinological anomalies and modifications in the Glu/GABA pathway. While the precise processes behind anxiety are not yet fully understood, existing research indicates that alterations in the Glu/GABA system may have a substantial impact (Saki et al. 2014). The empirical investigation carried out by Lacerda-Pinheiro et al. (2014) yielded substantiating evidence for the role of GABA in processes associated with anxiety. This was demonstrated by the activation of the GABA_A_ receptor by drugs with anxiolytic properties. In the study conducted by Luscher et al. (2011) empirical data was offered to support the notion that the etiology of depression and anxiety involves the concentration of GABA and the functioning of its receptors. Additionally, the successful management of anxiety and depressive disorders can be achieved by the use of antidepressant drugs that modulate the activity of monoaminergic neurotransmitters and GABAergic transmission. According to the study conducted by Soussan and Kjellgren (2016), it is postulated that GABA may elicit more favorable outcomes and result in less dependency. Multiple studies have demonstrated that *Lactobacillus rhamnosus* has the ability to regulate the expression of GABA receptors. Additionally, *Lactobacillus helveticus* and *Bifidobacterium longum* have been found to have antidepressant-like effects and alleviate anxiety symptoms in individuals diagnosed with depression.

### Cell cancer

GABA has become a crucial component in the domain of oncology, garnering considerable interest in recent scientific investigations due to its complex mechanistic functions in cancer therapy. The fundamental mechanism by which GABA impacts cancer therapeutics is through its interaction with a wide range of receptors, which triggers a succession of intricate intracellular signaling pathways. The regulation of critical cellular processes, including apoptosis and proliferation, is dependent on these pathways; these processes are essential for the progression and metastasis of cancer (Dou et al. 2023). When GABA binds to metabotropic (GABA-B) and ionotropic (GABA-A and GABA-C) receptors, a series of signaling cascades is initiated. These events have significant ramifications for the biology of cancer cells. It is worth noting that studies have demonstrated that activating GABA-B receptors inhibits adenylate cyclase, consequently resulting in a decrease in cyclic AMP (cAMP) concentrations. The decrease in cAMP is of utmost importance due to its influence on the functionality of protein kinase A (PKA), a kinase that has been linked to mechanisms of cell survival (Al-Wadei et al. 2009). Due to the correlation between GABA signaling and cell survival pathways, targeting GABAergic pathways in cancer treatment is a viable option.

Numerous studies that investigate the function of GABA in cancer cell proliferation have shed light on its ability to impede the development of particular cancer cell lines, including colorectal cancer cells (HT29). The observed inhibition is thought to be achieved via GABA receptor interactions that modulate cell cycle regulators; this may entail the downregulation of cyclins and cyclin-dependent kinases (CDKs), ultimately resulting in cell cycle arrest (Hydbring et al. 2016). In addition, intracellular calcium levels may be modulated as a consequence of the hyperpolarization of the cell membrane induced by the activation of ionotropic GABA receptors. Calcium signaling plays a critical role in numerous cellular processes, including those that promote the survival and proliferation of cancer cells; therefore, GABA is a crucial regulator in these pathways (Iizumi et al. 2022).

GABA exerts an influence that transcends its immediate impact on cancer cells and permeates the tumor microenvironment. According to Bao et al. (2023), it possesses the capability to regulate the conduct of stromal and immune cells present in the tumor microenvironment, thus exerting an impact on the progression of the tumor and the dissemination of its metastases. The function of GABA in angiogenesis, a critical process in tumor growth and metastasis, is of particular interest. Beheshtizadeh et al. (2023) have presented evidence that GABA might exert anti-angiogenic effects via the modulation of factors, including Vascular Endothelial Growth Factor (VEGF). Furthermore, the potential modulation of cell adhesion and migration processes by GABA, which may influence metastasis, presents novel opportunities for therapeutic development and research (Dahn et al. 2022). Therefore, given the complex and varied mechanisms by which GABA exerts its anti-cancer effects, it could be considered a viable therapeutic target in the field of oncology. Further investigation and development are warranted due to the comprehensive and prospective approach that GABA and it signaling pathways provide in modulating cell proliferation, angiogenesis, and interactions within the tumor microenvironment (Dong et al. 2023).

### Promoting growth hormone secretion

GABA primarily modulates the secretion of growth hormone (GH) via a cascade of neuroendocrine processes that involve the hypothalamus–pituitary axis (HPA). Beginning with GABAergic neurons in the hypothalamus, where GABA regulates the secretion of growth hormone-releasing hormone (GHRH), the process commences. After being secreted, GHRH reaches the anterior pituitary gland, where it instructs it to release GH into the circulation. Concurrently, GABA facilitates the suppression of somatostatin, a hormone that typically inhibits the release of growth hormone. GABA indirectly promotes a milieu that is more favorable for the secretion of GH by impeding somatostatin (Powers 2012). Furthermore, the effect of GABA extends beyond the hypothalamus. Additionally, the pituitary gland contains GABA receptors, which implies that GABA might stimulate these receptors directly, resulting in increased GH secretion (Cavagnini et al. 1982). This direct pathway provides a secondary route by which GABA can increase GH levels, distinct from its hypothalamic effect. In addition to its primary function, GABA exerts an impact on various neurotransmitter systems, including serotonin and dopamine, which in turn regulate the secretion of GH. This additional factor introduces a level of intricacy to the manner in which GABA regulates GH levels. Scientific evidence substantiates these mechanisms. Research has shown that the administration of GABA supplements can result in substantial elevations in GH levels. As an illustration, the consumption of 3 g of GABA resulted in a 400% increase in GH concentrations; furthermore, GH levels increased significantly when combined with resistance training or whey protein (Powers et al. 2008; Sakashita et al. 2019). Furthermore, clinical trials have demonstrated the potential of GABA-enriched foods, such as fermented sea tangle, to augment GH production and enhance body composition (Choi et al. 2016). However, these results also emphasize the temporary and GH secretion-responsible nature of GABA’s effects, which are dose-dependent. This underscores the complex and ever-changing interaction between neuroendocrine factors that govern growth hormones.

## GABA-producing strains and metabolic pathway

Microbial fermentation has played a great role in GABA production. *Lactobacillus* (Yogeswara et al. 2020a), *Escherichia coli* (Yuan et al. 2019) and *Corynebacterium glutamicum* (Baritugo et al. 2018) are some of the natural microorganisms that contributed to producing GABA. Regardless, GABA production is relatively low when using microorganisms and cannot intensify for commercialization.

### Lactic acid bacteria

LAB are significant GABA-producing microbes because to the diverse production features and probiotic benefits that they possess. Several LAB strains with the potential to produce GABA have been identified from traditionally fermented foods such as cheese, kimchi, paocai, yoghurt, and fermented soy beans, amongst others (Cui et al. 2020). Cocci, or rod-shaped, gram-positive, acid-tolerant, and non-sporing LAB is the key microorganism in GABA production (Yogeswara et al. 2020b). Furthermore, various *Lactobacillus* species, including, *L. brevis* (Mancini et al. 2019; Seo et al. 2013a), *L. buchneri* (Mugampoza et al. 2020), *L. delbrueckii* subs. *Bulgaricus* (Câmara et al. 2019; Gangaraju et al. 2014), *L. plantarum* (Zhuang et al. 2018), and *L. helveticus* (Li et al. 2020) have demonstrated significant potential in GABA biosynthesis and strong endogenous GAD activity. Moreover, *Streptococcus thermophilus* and *Lactococcus lactis* have the strength for producing GABA-rich milk products. Furthermore, several species from the genus *Bifidobacterium*, *Enterococcus*, *Leuconostoc*, *Pediococcus*, *Propionibacterium*, and *Weissella* have the resilience to produce GABA (Diana et al. 2014; Franciosi et al. 2015). Recent studies have shown that certain bacterial strains, mainly from the genera *Lactobacillus* and *Bifidobacterium*, influence the functioning of the central nervous system, leading to changes in behavior, nociception and cognitive abilities (Yunes et al. 2020). According to the study of Li et al., *Lactobacillus brevis* is the most widely isolated species among GABA-producing LAB and has the highest production and GABA titer under appropriate pH, temperature, and time conditions. For example, *Lactobacillus brevis* NCL912, which was isolated from Chinese paocai, was able to produce GABA at a concentration of 103.72 g/L. Optimizing culture conditions was critical for achieving this high GABA concentration. The optimal temperature was determined to be 32 °C, which balanced cell growth and efficient GABA synthesis. The ideal pH level was found to be 5.0, which aligned with the activity range of *Lactobacillus* GAD enzymes while maintaining their activity. Additionally, a fermentation period of 48 h was identified as optimal, with rapid GABA production observed in the initial 36 h (Li et al. 2010; Wu and Shah 2017). In comparison to other LAB species, *Lb. brevis* has been proven in a significant number of experiments to be capable of producing a greater quantity of GABA. The maximum yield of GABA that has been achieved by *Lb. brevis* so far is 205 g/L (Wang et al. 2018b). The recent GABA-forming strain *L. futsaii* CS3 isolated from fermented shrimp was able to convert 25 mg/mL of MSG to GABA with a yield of more than 99% within 72 h (Sanchart et al. 2016, 2017). Moreover, the novel GABA-producing *Enterococcus avium* isolated from Korean traditional fermented anchovy and shrimp was able to produce 18.47 mg/mL of GABA within 48 h in the MSG medium (Lee et al. 2017).

Optimization of culture conditions for various GABA-producing strains is much crucial in higher GABA yield. At initial pH 5.0, *L. brevis* NCL912, and *L. buchneri* have produced the maximum GABA yield (Cho et al. 2011; Li et al. 2010). Though *L. brevis* 100 yielded the highest GABA titer at pH 3.5 during the fermentation of black raspberry juice. Henceforth, the optimal pH of LAB for GABA production ranged from 3.5 to 5.0 (Kim et al. 2009). Besides, the optimum temperature for the highest GABA titer for most of the microorganisms is in the range of 25–37 °C (Villegas et al. 2016). The optimal MSG concentration for GABA production is varied according to the strains. There was no significant change in the range of 10–20 g/L MSG in *S. thermipillus* Y2 for GABA production (Yang et al. 2008). The precise MSG concentration for *L. brevis* CRL 1942 was 100 mM (Villegas et al. 2016). However, in the concentration of 100 mM of L-MSG, the GABA titer was 721.35 mM, which is 7.7 times higher than that without MSG during the fermentation of *L. planatarum* CGMCC 1.2437^T^ (Zhuang et al. 2018). So, despite the fact that numerous GABA-producing LAB strains have already been separated and identified, additional study on the isolation and characterization of the LAB is required since different types of GABA-producing LAB are crucial for the food sector (Komatsuzaki et al. 2005).

### *Escherichia coli*

*Escherichia coli (E. coli)* bacteria can survive in an extremely acidic environment because of their intracellular glutamate-dependent acid resistance system (Ma et al. 2012). Researchers have used this benefit for the production of GABA by *E. coli*. In the l-glutamate catabolic pathway in *E. coli* GABA can be synthesized by catalyzation of glutamate decarboxylases (GADs). There are six subunits in *E. coli* GAD and every subunit consists of pyridoxal phosphate (PLP) as the coenzyme and PLP-dependent is 52.6 kDa. GAD has a decisive substrate selectivity for l-glutamate and its optimal pH is about 3.8 (Wang et al. 2011). In *E. coli* BL21, a new GAD from *Lb. senmaizukei* was expressed, yielding 4.8 g/L GABA from l-glutamic acid with PLP. The synthetic protein scaffold enabled the co-expression of gadA and gadC, increasing the GABA titer to 5.65 g/L with a 93% conversion rate (Le Vo et al. 2012, 2013).

The acid resistance system of *E. coli* consists of three key proteins; two glutamate decarboxylase isozymes (GadA and GadB) and the glutamate/GABA anti-porter (GadC). The ability to resist an extremely acidic environment of pH 2.5 or less and generally limited to stationary-phase cells is known as acid resistance in *E. coli*. Previous studies have reported that there are three acid-resistance systems in *E. coli* and among them, the third acid-resistance system is required for encoding gadA or gadB and GABA antiporter gadC (Merchel Piovesan Pereira et al. 2021). As a result of the ability of *E. coli* can survive under extremely acidic environments GadC can shift into the cytoplasm while GadA and GadB catalyze the decarboxylation reaction and they consumed hydrogen ions to produce GABA (Ma et al. 2012).

Researchers have worked hard to develop modified *E. coli* strains that can generate GABA from renewable substrates without the need of precursors. A high concentration of 4.8 g/L GABA was generated by an engineered strain of *E. coli*. This was the maximum amount of glucose produced in modified *E. coli* without the use of precursors. By utilizing advanced genetic engineering techniques, researchers successfully modified *E. coli* to express a specific set of genes: gadB, gadC, icdA, gdhA, and dr1558. This strategic alteration markedly enhanced GABA synthesis, achieving an impressive yield of 6.16 g/L from glucose in an acidic environment with a pH of approximately 5.0 (Park et al. 2020). In addition, by shutting off the opposing metabolic pathways through gene deletion, the final GABA titer from glucose was raised to 1.3 g/L (Lee et al. 2015).

To further improve GABA biosynthesis, several genetic engineering techniques were applied to *E. coli BW25113*, including conditional interruption of the TCA and glyoxylate cycles, engineering of the GABA production pathway (including a bypass for precursor metabolite supply, and upregulation of GABA transporter) (Soma et al. 2017). According to different research, the gadB mutant gene’s expression has the ability to convert glycerol into GABA. Even though the GABA titer in the modified *E. coli* was just 0.98 g/L, it nevertheless provides a helpful way to use the waste product created by the biodiesel industry (Hou and Kang 2018). In a groundbreaking study, other researchers successfully enhanced GABA production in *Escherichia coli* Nissle 1917 (EcN) through genetic engineering. The approach involved expressing the gadB gene, responsible for GABA synthesis, in EcN using the stable vector pMT1. This resulted in a remarkable GABA production level of 17.9 g/L in an antibiotic-free system. The study also investigated the stability of various plasmids in EcNP, a plasmid-free derivative of EcN. The pMT1-J-GadB plasmid demonstrated a stability rate of 36% after four passages, significantly outperforming other plasmids such as pSU-J-GadB, which lost all stability. This highlights the superior resilience and efficiency of the pMT1 vector in GABA production. Furthermore, the study emphasized the importance of promoter selection and plasmid copy number (PCN) in protein expression. The J23100 promoter proved to be the most effective for driving gene expression in both EcN and EcNP, indicating that optimizing these factors can significantly enhance the efficiency of GABA production in genetically engineered *E. coli* Nissle strains (Lan et al. 2021). This research represents a significant breakthrough in the field of probiotic genetic engineering for improved GABA synthesis, opening up new possibilities for the development of innovative probiotics with enhanced health benefits.

### *Corynebacterium glutamicum*

*Corynebacterium glutamicum (C. glutamicum)* is a popular choice for a microbial cell factory (MCF) to produce l-glutamic acid, which is a necessary precursor in the biosynthesis of GABA. As well as being a good chassis bacterium, it is also a GRAS microbe that possesses the biosynthetic gene for the GABAPCg transporter, an essential enzyme for the absorption and production of GABA. By expressing genes from Lb. brevis LB85 and *C. glutamicum* ATCC 13032, *C. glutamicum* was created for GABA production without the inclusion of the precursors (MSG or l-glutamate). With productivity of 0.030 g/L in shaking flasks, it generated 2.15 g/L GABA from 160 g/L glucose, which was less than what LAB produced (Shi and Li 2011). The 2-oxoglutarate dehydrogenase (ODHC) complex subunit E1, which is encoded by the odhA gene, lowers the metabolic flow to l-glutamate in *C. glutamicum*. The phosphoenolpyruvate-rcarboxylase (PEPC), which is encoded by the ppc gene, is helpful for *C. glutamicum*’s (26.3 g/L) GABA biosynthesis (Tsuge and Matsuzawa 2021). Baritugo et al. have investigated recombinant *C. glutamicum* strain (co-expression of the gadB mutant gene and the xylAB gene-encoding xylose isomerase and glucokinase) produced 35.47 g/L of GABA at the medium of glucose and xylose as the substrate. It concluded that two ample fermentative sugars in lignocellulose such as glucose and xylose can be co-utilized by *C. glutamicum* (Son et al. 2022).

Inconsistencies in pH prevent *Corynebacterium glutamicum* from producing enough GABA. Recombinant cultures that expressed mutant GADs from *Lactococcus lactis*, *Lactobacillus senmaizukei*, and *Escherichia coli* shown improved pH stability and adaptability at 7.0. Son et al. (2022) studied the synthesis of GABA at pH values of 5.0, 6.0, and 7.0. Batch fermentations of *C. glutamicum* H36EcGADmut (40.3 and 39.3 g L^−1^), H36LlGAD (42.5 and 41.1 g L^−1^), and H36LsGAD (41.6 and 40.2 g L^−1^) with 100 g L^−1^ glucose produced higher GABA titers and yields at pH 6.0 and pH 7.0. However, a CRISPR/Cas9-coupled recombination method has been created for effective cure. Random mutagenesis and antiquated recombination procedures continue to be used in the genome engineering of *C. glutamicum*. Important genes (Ncgl1221, gabT, and gabP), as well as the expression of the gadB2 gene, were used (Cho et al. 2017).

### *Bacillus*

*Bacillus* species are known to have a crucial role in facilitating natural GABA production. Among the various Bacillus species, *Bacillus subtilis* and *Bacillus amyloliquefaciens* have shown promising results in generating significant GABA concentrations in recent years (Wang et al. 2019; Asun et al. 2022).

Recent studies have demonstrated the remarkable ability of *Bacillus subtilis* BBEL02 to produce GABA, with a maximum concentration of 10.9 g/L achieved through the utilization of industrial waste as a feedstock. This sustainable and economical approach highlights the strain’s capability to thrive on affordable substrates, rendering it an attractive option for large-scale production. Further advancements in the fermentation process, involving a 2-L scale and optimized conditions, resulted in an increased GABA concentration of 12.5 g/L, maintained at an optimal 80% dissolved oxygen level. Notably, the use of soybean hydrolysate as a nitrogen source proved both cost-effective and efficient, reinforcing its potential for widespread implementation (Asun et al. 2022). *Bacillus subtilis* ATCC 6051 stands out among six probiotic bacteria, boasting an impressive 19.74 g/L GABA output under optimal conditions (30 °C, pH 8.0) using a medium comprised of 11.481 g/L potato starch, 60 g/L peptone, 5 g/L NaCl, and 2.5 g/L K2HPO4. Additionally, 11.825 g/L of sodium l-glutamate was added to the medium after 48 h to ensure efficient formation of GABA. This result underscores the efficacy of *Bacillus* species in GABA production (Wang et al. 2019). Genetic engineering plays a vital role in enhancing GABA production in *Bacillus subtilis*. By introducing the glutamate decarboxylase gene from *Streptococcus salivarius*, a significant increase in GABA production was achieved, culminating in a remarkable rate of 512.9 µmol/h/g. This modification enabled the bacterium to produce up to 5.26 g/L of GABA within just 12 h, surpassing previous yields (Zhang et al. 2014a). The potential of *Bacillus* species extends beyond conventional fermentation methods. The expression of glutamate decarboxylase genes from *Bacillus* spp. in *E. coli* resulted in a high molar conversion rate of 98.6% to GABA, showcasing the effectiveness of engineered microbial strains. Specifically, the GADZ11 gene from *Bacillus* spp., when expressed in *E. coli*, displayed the highest efficiency, attaining a 98.6% molar conversion rate to GABA in merely 14 h. This achievement highlights the tremendous capacity of genetic manipulation to optimize microbial strains for large-scale industrial production (Sun et al. 2021). Furthermore*, Bacillus methanolicus*, when engineered to express glutamate decarboxylase genes, successfully produced 9 g/L of GABA via a novel two-phase production strategy that utilizes methanol as a non-food raw material. This innovative approach illuminates the versatility and adaptability of Bacillus species in biotechnological processes (Irla et al. 2017).

Regarding the production of GABA by microorganisms, distinct metabolic pathways and regulatory mechanisms are observed in each strain. LAB, which includes *Bifidobacterium* and *Lactobacillus* strains, produces GABA via the glutamate decarboxylase pathway. These bacteria operate most efficiently in anaerobic and mildly acidic environments. The bacterial response to environmental stress, specifically acid stress, is intricately linked to GABA synthesis along this pathway. As a result, GABA production is integrated with cellular health and growth as a whole (Cui et al. 2020; Yogeswara et al. 2020b). On the other hand, *E. coli* employs a glutamate-dependent acid resistance mechanism that comprises the glutamate/GABA antiporter GadC and the enzymes GadA and GadB. The metabolic regulation of this system is predominantly influenced by acidic surroundings; therefore, substantial genetic engineering efforts are required to optimize GABA yields; thus, genetic modifications and metabolic regulation are directly linked (Wang et al. 2011; Le Vo et al. 2012). Genetic modification of *C. glutamicum*, which was previously employed for the synthesis of l-glutamic acid, to generate GABA through modification of the tricarboxylic acid (TCA) cycle and upregulation of gadB indicates that metabolic regulation in *C. glutamicum* is dependent on genetic engineering (Shi and Li 2011; Tsuge and Matsuzawa 2021). *Bacillus* species, including *Bacillus subtilis* and *Bacillus amyloliquefaciens*, produce GABA naturally as part of their amino acid metabolism. Specific strains of Bacillus demonstrate increased GABA production under particular fermentation conditions, indicating that the composition of external media may affect the regulation of their metabolic processes (Wang et al. 2019; Asun et al. 2022). In conclusion, LAB is recognized for its naturally regulated and efficient GABA production pathway, which renders it well-suited for applications in the food industry. On the contrary, *Bacillus* species, *E. coli*, and *C. glutamicum*, which necessitate significant genetic modifications, possess inherent GABA production capabilities and offer substantial potential for improved GABA production in industrial environments. It is imperative to comprehend these varied metabolic pathways and regulatory mechanisms in order to optimize the proliferation of cells and the production of GABA in these microorganisms.

### Metabolic pathways of GABA

The synthesis of GABA, which begins with the conversion of glutamate, entails a complex series of enzymatic reactions. The process proceeds with the decarboxylation step, which is carried out by glutamate decarboxylase (GAD), an enzyme that comes in two different forms (GAD65 and GAD67). The removal of the carboxyl group requires the cofactor PLP from vitamin B6 (Yuan et al. 2019). This synthesis pathway, mediated by the enzyme (GAD), results in the production of GABA, which plays a crucial role in regulating neuronal excitability and the overall stability of the neural network. Furthermore, glutaminase and glutamine synthetase play crucial functions in glutamic acid, glutamine, and ammonia homeostasis in the brain, which is intimately connected to GABA biosynthesis (Andersen and Schousboe 2023).

#### GABA shunt

**Fig. 2:**
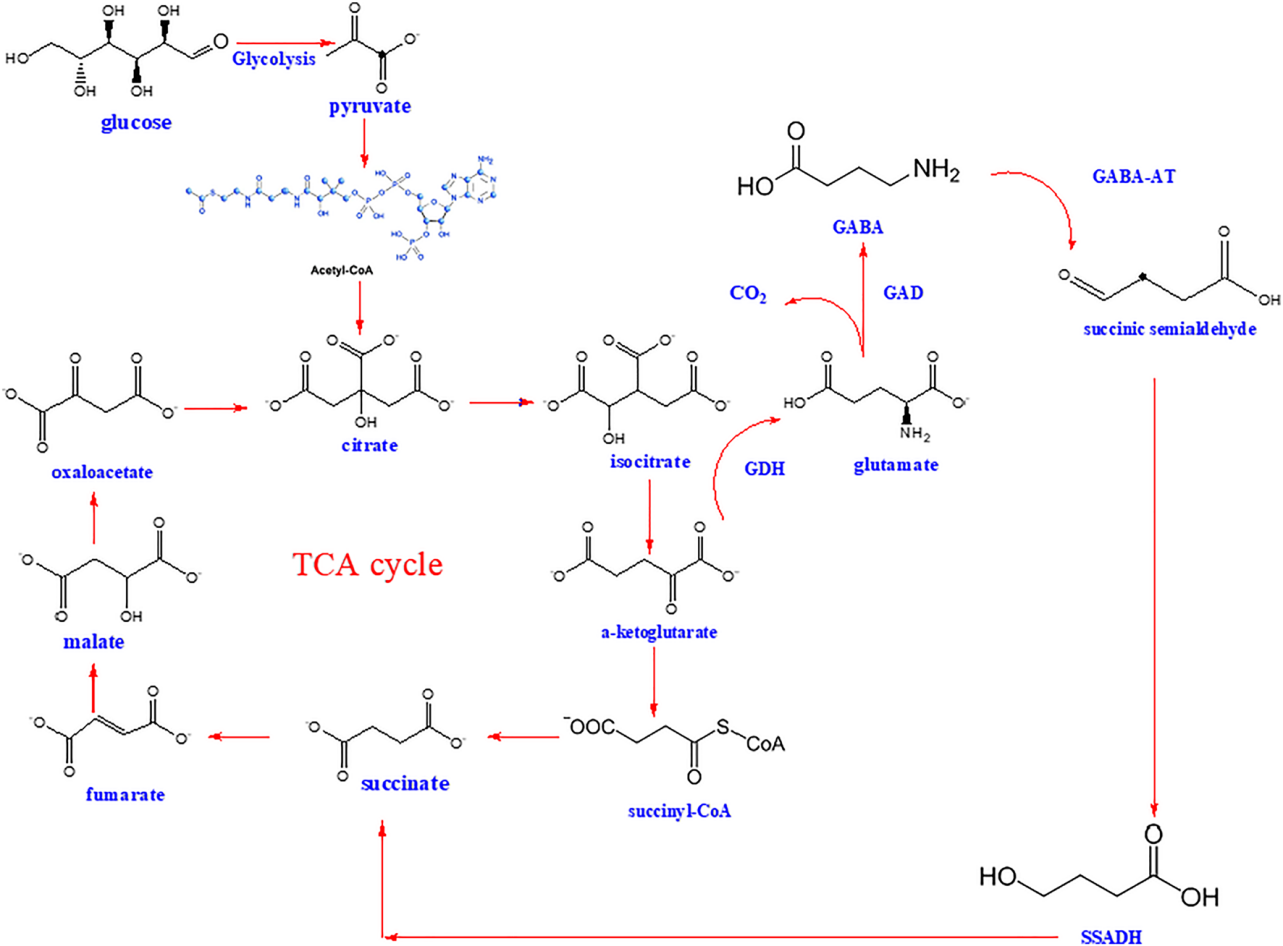
Metabolic pathway of GABA production from the TCA cycle. *TCA* tricarboxylic acid cycle, *GDH* glutamate dehydrogenase, *GAD* glutamate decarboxylase, *GABA* γ-aminobutyric acid, *GABA-AT* γ-aminobutyric acid aminotransferase, *SSADH* succinic semialdehyde dehydrogenase

The main GABA route involves the conversion of alpha-ketoglutarate produced by the TCA cycle to succinate via glutamate, GABA, and succinic semialdehyde (Sarasa et al. 2020). This mechanism, often referred to as the GABA shunt, is shared by prokaryotes and eukaryotes. In 1970, a study on guinea pig cells led to the first description of the GABA shunt pathway (Hammond et al. 1970). The primary function of GABA shunt is GABA production. GABA was also synthesized via polyamine (putrescine and spermidine) degradation, and it occurs via a non-enzymatic reaction from proline under oxidative stress (Fait et al. 2008; Shelp et al. 2012; Signorelli et al. 2015). The initial stage in the GABA breakdown process, known as succinic semialdehyde (SSA) interconversion, can be catalyzed by GABA transaminase (GABA-T). SSA is converted to succinic acid (SA) by semialdehyde dehydrogenase (SSADH), which is followed by the Krebs’s cycle, where SA is dehydrated. The existence of SSADH, which is quite active, prevents SSA from actually reversing the production of GABA, despite the fact that it theoretically might (Le Vo et al. 2012; Shelp et al. 1999). The glycolysis process transforms glucose into pyruvate; then, pyruvate is converted into acetyl-CoA, which combines with oxaloacetate to produce citrate, which enters the TCA cycle. Citrate is converted to isocitrate and alpha-ketoglutarate, which can then be turned to GABA via glutamic acid dehydrogenase (GDH) and GAD by a variety of bacteria (Chen et al. 2019). GABA may be degraded by gamma-aminobutyric acid aminotransferase (GABA-AT) and semialdehyde dehydrogenase in the GABA shunt. GABA is converted to succinate by these enzymes, which subsequently enters the TCA cycle. The first is a reversible process by GABA-AT that creates succinic semialdehyde (SSA), and the second is an SSADH reaction that converts SSA to succinate. As the TCA cycle occurs in the mitochondria, whereas the cytosol forms GABA from glutamate; as GABA is converted by GABA-AT and SSADH, it returns to the mitochondria (Fig. 2) (Bown and Shelp 2020).

### Enzymatic preparation of GABA

**Fig. 3:**
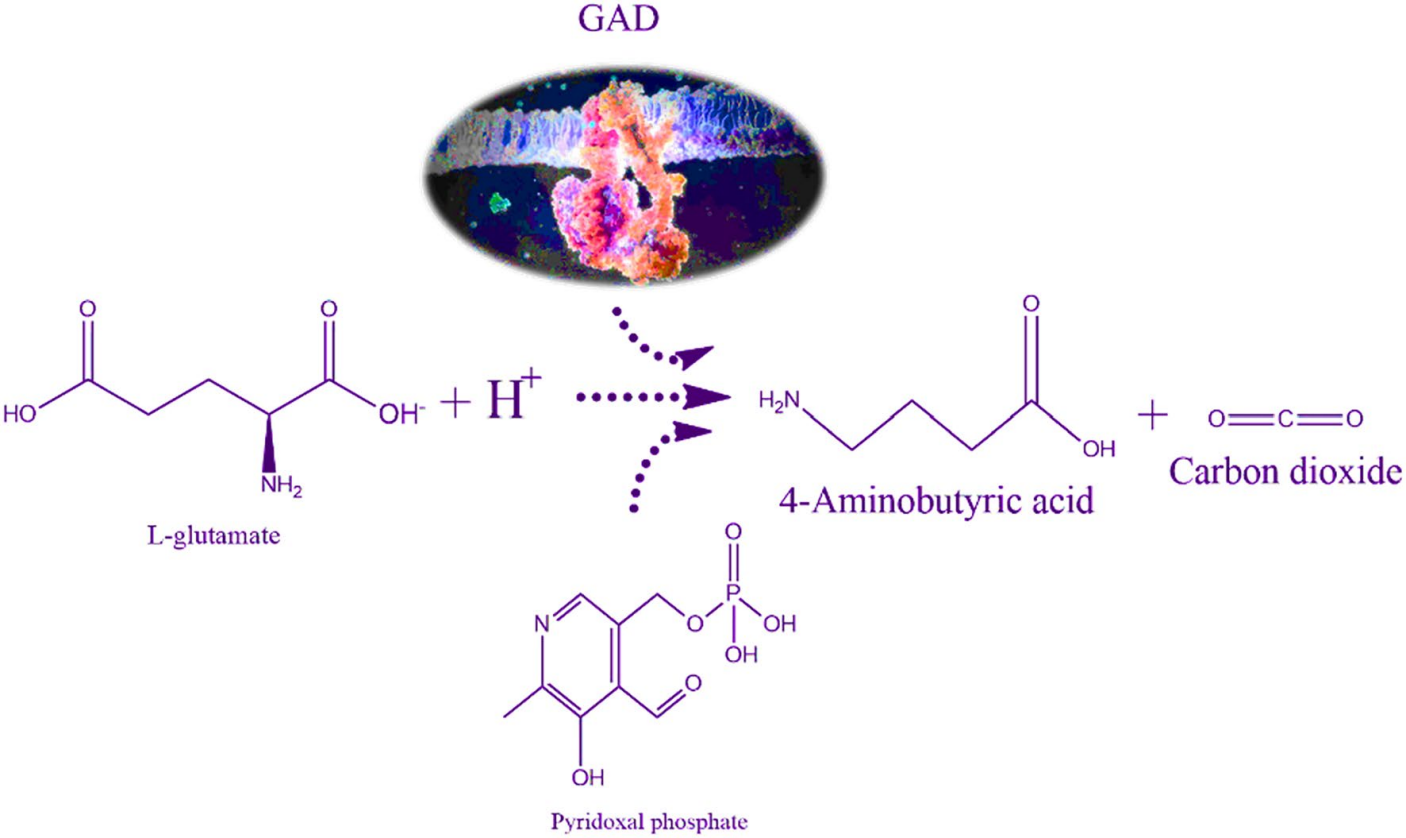
Decarboxylation of l-glutamate to GABA by glutamate decarboxylase, *PLP* pyridoxal-5′-phosphate

The GABA shunt involves various enzyme types, with the transamination process, catalyzed by glutamate dehydrogenase (GDH), being the first step in synthesizing glutamate from alpha-ketoglutarate. The subsequent phase involves glutamate decarboxylase, converting glutamate to GABA while consuming a proton and producing CO_2_. Glutamate decarboxylase (GAD), found in organisms across all life kingdoms, is a rate-limiting enzyme in GABA production, requiring the cofactor pyridoxal phosphate (PLP) for its function (Roberts and Kuriyama 1968). The α-decarboxylation of l-glutamate, primarily catalyzed by GAD with PLP as a cofactor, is the main metabolic pathway for GABA biosynthesis in most GABA-producing microorganisms (Yogeswara et al. 2020b), as illustrated in Fig. 3. Additionally, microorganisms can biosynthesize GABA through the degradation of compounds like putrescine, polyamine, or ornithine (Yu et al. 2019b). GABA transaminase, the third enzyme in the GABA shunt, facilitates GABA catabolism, leading to succinic semialdehyde (SSA). SSA is then converted to succinate by succinic semialdehyde dehydrogenase, integrating into the tricarboxylic acid (TCA) cycle and serving as an electron donor to the mitochondrial electron transport chain (Sarasa et al. 2020). GAD typically exhibits higher activity and stability in acidic conditions due to favorable protonation states of PLP and glutamate’s amino group, though this pH dependency varies among GAD variants. For example, *E. coli*’s GAD is optimal at pH 4.8, while *Lactobacillus plantarum*’s GAD functions best at pH 5.5 (Yogeswara et al. 2020a. Advances in biotechnology have allowed for the modification of GADs to function effectively in neutral or alkaline pH. For instance, the overexpressed and purified GAD from *Mycobacterium smegmatis* (GADMSM) showed optimal activity at pH 5.4, retaining significant activity at pH 6.2. Mutants like GADMSMΔC displayed high activity across a pH range of 5.0–7.0, with considerable activity retention at pH 7.0 (Li et al. 2022b). Similar engineering efforts have been made with GADs from *Lactococcus lactis* (Son et al. 2022) and *E. coli* (Alexander et al. 2023), enhancing their activity and stability at neutral or alkaline pH for efficient GABA production.

In one study, the GABA production of thirty lactic acid bacteria isolates from fermented foods was evaluated, with *Lactobacillus plantarum* strains FNCC 343 and FNCC 260 standing out. *L. plantarum* FNCC 260’s GABA production significantly increased with MSG addition, from 809.2 to 1226.5 mg/L. The strain’s gadB gene was effectively introduced into *E. coli*, leading to a more efficient enzymatic conversion of MSG to GABA than traditional fermentation methods (Yogeswara et al. 2020a). Another study successfully enhanced GABA synthesis in *E. coli* by overexpressing GadB and applying a − 20 °C cold treatment, achieving a yield of 46.9 g/L (Xue et al. 2021). These studies represent significant progress in GABA biosynthesis, improving scalability and efficiency, which are crucial for its application in pharmaceutical and food industries.

## Strategies to enhance the microbial production of GABA

GABA may currently be produced using a number of techniques, such as chemical synthesis, plant enrichment, enzymatic activity, and microbial production. It cannot be synthesized chemically due to safety concerns, but it can be made enzymatically using l-glutamate and GAD. Microbial production is preferred because it employs renewable resources, emits less pollutants into the environment, and complies with green regulations in the food and pharmaceutical industries. GABA may be produced by modified LAB strains using low-cost, renewable resources such lignocellulosic biomass, glucose, and glycerol (Luo et al. 2021). In the current state, the genome-scale metabolic model (GSMM) provides a framework for examining strain metabolic functions, aiding in the design of LAB metabolic systems and assisting in the development of effective strategies for strain enhancement. GABA synthesis by LAB species may be strain-specific, and fermentation conditions have a big influence on production (Luo et al. 2021).

### Optimizing factors affecting GABA fermentation production

#### pH

The value of pH is an essential component in the production of GABA by LAB. Not only does it have an effect on the development of bacteria, but it also has an impact on the activity of GAD (Cho et al. 2007; Kim et al. 2009; Komatsuzaki et al. 2005; Li et al. 2010; Yang et al. 2008). According to certain research (Li et al. 2010) the initial pH of the fermentation medium had an impact on the production of GABA. At an initial pH of 5.0, *Lb. brevis* NCL912 could synthesize the most GABA. *Lactobacillus paracasei* NFRI 7415 produced GABA at an initial pH of 5.0, which was substantially higher than the starting pHs of 4.0 and 6.0 (Wang et al. 2021) for *Lb. buchneri* to synthesize GABA, a pH of 5.0 is also ideal in the beginning (Cho et al. 2007). However, during the fermentation of black raspberry juice, Lb. brevis GABA100 produced the greatest amount of GABA at an initial pH of 3.5 (Kim et al. 2009). As a result, the ideal pH range for fermenting microorganisms ranges from pH 3.5 to pH 5.0, depending on the various characteristics of GADs. In order to produce GABA effectively, a low pH must be maintained. Interestingly, *S. thermophilus* Y2 cells may greatly enhance GABA synthesis by raising the pH of the growth medium to 4.5 once every 12 h (Komatsuzaki et al. 2005; Yang et al. 2008). Another, at a pH of 7.1, *L. lactis* produced the maximum GABA (7.2 g/L), but GABA production dropped at pH values above 8 (Lu et al. 2009). Since the pH of the fermentation medium changes over time and impacts the final concentration of GABA, early media pH adjustment is required for the optimal pH.

#### Temperature

The temperature of the culture has a significant role in the generation of GABA and bacterial growth. The ideal temperature for *Lb. brevis* CRL 1942 to produce GABA was 37 °C. *Lb. brevis* NCL912’s ability to produce GABA was influenced by the temperature and cell density of the culture. Temperature-related growth gained peak at 35 °C and then declines over time. At 30 °C and 35 °C, *Lb. plantarum* DSM19463 produces the most GABA (Di Cagno et al. 2010). Moreover, *Lactobacillus brevis* GAD and *Lactobacillus brevis* CGMCC 1306 are found to exhibit their best performance at 30 °C and 37 °C, respectively. In a study by Kim et al. (2009), *Lactobacillus brevis* GABA 100, fermenting black raspberry juice, achieved its highest GABA production on the 12th day of fermentation at 30 °C. In contrast, Park et al. (2006) found that *Streptococcus salivarius* subsp. thermophilus reaches its peak GABA production at an ideal temperature of 34 °C. Additionally, the study conducted by Wang et al. (2021) provides valuable insights into the effect of temperature on GABA production, specifically in relation to the bacterial strain *Lb. paracasei* NFRI 7415. The research demonstrated that this particular strain achieved peak GABA production at a temperature of 37 °C, with a discernible decline in cell growth occurring at temperatures around 43 °C. These findings support the broader understanding that GABA yield is optimized within a temperature range of 25 °C to 40 °C. This study not only reinforces the critical role of temperature in GABA synthesis but also highlights the importance of carefully optimizing fermentation conditions to achieve maximum GABA production in different bacterial strains.

#### Carbon and nitrogen sources

LAB synthesis of GABA via fermentation is influenced by the composition of the media, including carbon and nitrogen sources. Organisms need carbon as the primary component for growth. Other than acting as an accelerator for the creation of bioactive chemicals through the secondary metabolism pathway, carbon’s primary job was to build cell biomass. Rayavarapu et al. (2021) recently evaluated the influence of different carbon (glucose, sucrose, lactose, and maltose) and nitrogen (MSG, peptone, yeast extract, beef extract) sources on GABA synthesis by *L. fermentum*. Besides, GABA synthesis from carbon and nitrogen sources is significantly influenced by glucose and monosodium glutamate (MSG), respectively. GABA yield and cell density increased as the glucose concentration increased from 0.5 to 1.0%, while 2% glucose significantly reduced GABA synthesis. As a result high glucose levels promote cell shrinking, which inhibits growth and GABA synthesis (Stier and Kulozik 2020). The effects of several carbon sources (glucose, saccharose, xylose) and nitrogen sources (peptone, K2HPO4, l-sodium glutamate) on the synthesis of gamma-aminobutyric acid in mulberry leaf powder were investigated by Zhong et al. (2019). The researchers observed that the combination of saccharose and K2HPO4 resulted in the highest synthesis of GABA. Additionally, they found that carbon sources consisting of 1% glucose and 1% peptone were the most efficient in promoting GABA production. According to research by Kim et al. (2021) on the influence of synthetic medium composition on GABA production in *L. plantarum* KCTC 3103, the greatest GABA content (0.60 g/L) was realized after 72 h while utilizing glucose as the carbon source and 10 g/L yeast extract as the nitrogen source. Yeast extracts were shown to be the best nitrogen sources, increasing the sources and amounts of nitrogen while simultaneously promoting GABA production.

#### Impact of fermentation time on GABA yield

The quantity of GABA generated is significantly influenced by the time of incubation. It has been shown that for LAB strains, GAD conversion to GABA began in the late logarithmic growth phase and peaked in the stationary phase (Hayakawa et al. 2004). The highest GABA production was achieved on the 15th day when black raspberry juice fermented with *Lb. brevis* GABA 100 at 25 °C and 37 °C, while the highest was on the 12th day when fermented at pH 3.5 and 30 °C. The maximum GABA output was achieved by adding MSG over 6 to 96 h, indicating a significant difference in GABA yield between different durations of MSG addition in *L. lactis* fermentation (Lu et al. 2009). GABA production is affected by fermentation duration, with longer periods resulting in greater amounts. To create 4.83 mM and 60 mM GABA, *L. plantarum* and *L. paracasei* need 72 and 144 h, respectively. *L. lactis* needs 24 h in mulberry beer to produce 1031 mg/kg (Zhang et al. 2020). GAD converts to GABA optimally in the stationary phase and during the late logarithmic growth phase. *L. fermentum* generates GABA slowly over 24 h, reaching a peak concentration of 4.6 g L1 after 48 h. *L. brevis* RK03 yields the most GABA after 88 h of fermentation, whereas *L. plantarum* and *L. paracasei* release the most GABA after 72 and 144 h, respectively (Pannerchelvan et al. 2023).

### Exploring diverse fermentation techniques

As mentioned before microbial synthesis has gained much attention in GABA production over chemical synthesis based on several factors. In fact, genetic engineering (Lan et al. 2021), two-stage fermentation (Kim et al. 2021), co-culture technique (Wang et al. 2019), fed-batch fermentation (Thongruck and Maneerat 2023), and enhancement of nutrition (Cataldo et al. 2020) are some common methods recently used in the biotechnology field. Herein (Table 2) summarized some applicable examples of common strategies. Among these strategies two-stage fermentation and co-culture techniques have particular advantages towards GABA production. Therefore, detailed explanations with examples of two-stage fermentation and co-culture techniques are mentioned below:

**Table 2:** The improvement of GABA production by different strategies

Strategy	Strains	pH	Temp (°C)	Time (h)	Substrates	Titer	Productivity	References
Genetic engineering	*Lactobacillus brevis* NRA6	4.5	37	48	72.75 g/L MSG	43.65 g/L	1.70 g/L/h	Lyu et al. (2017)
	*L. lactis* NZ9000/pNZ8148-*gadBC*	6.2	30	36	60 g/L L-MSG	25.61 g/L	0.7114 g/L/h	Lyu et al. (2020)
	*Lactobacillus sakei* B2-16	6.0	30	48	7% food-grade MSG	265.3 mM	0.57 g/L/h	Kook et al. (2010)
	*C. glutamicum* ATCC13032	6.5	30	72	10 g/L by feeding 500 g/L glycerol	45.6 g/L	0.633 g/L/h	Wei et al. (2022)
Two-stage fermentation	*C. tyrobutyricum* ATCC25755	4.5	37	60–84	588.52 g/L L-Glu and 90 g/L and 120 g/L glucose	400.32 g/L	36.39 g/L/h	Liu et al. (2023)
	*L. plantarum* KCTC 3103	6.0	30	60	Rice bran, ascorbic acid, MSG	0.67 g/L	0.0112 g/L/h	Kim et al. (2021)
	*Lactobacillus brevis* CGMCC 1306	5.0	35	72	20 g L-MSG	94.7394 g/L	1.316 g/L/h	Chunlong et al. (2013)
Co-culture fermentation	*Lactobacillus brevis* NPS-QW	4.5–6.4	37	48	1.5–2.0 g/L MSG	0.9 g/L	0.01875 g/L/h	Xiao and Shah (2021)
	*Bacillus subtilis* ATCC 6051	8.0	30	120	5 g/L sodium l-glutamate	19.74 g/L	0.1645 g/L/h	Wang et al. (2019)
	*Lacticaseibacillus rhamnonus* or *Lacticaseibacillus paracasei*	4.0–5.0	40	48	249.31 mg L^−1^ MSG	185.81 ± 24.0 and 319.72 ± 27.15 mg L^−1^	0.00387 g/L/h and 0.00666 g/L/h	Galli et al. (2022)
Fed batch fermentation	*Corynebacterium glutamicum* XW6-16	7.0	30	72	Glucose	77.6 g/L	1.21 g/L/h	Wen and Bao (2021)
	*L. futsaii* CS3	5.0	37	72	3.47% (w/v) cane sugar, 3.84% (w/v) tuna condensate and 10.77% (w/v) MSG	23.01 g/L	0.3196 g/L/h	Thongruck and Maneerat (2023)
	*C. glutamicum* CGY705	5.2–5.3	30	78	L-Glu, 0 ± 5 g/L Glucose	33.17 g/L	0.4252 g/L/h	Yao et al. (2023)
Enhancement of nutrition (carbohydrate fermentation)	*Lactobacillus brevis* CRL 2013	4–7.25	30	72	Xylose, ribose, glucose, galactose, MSG	27.33 g/L	0.380 g/L/h	Cataldo et al. (2020)
	*Lactobacillus plantarum* HU-C2W	4.90	37	40	Litchi juice	1.34 g/L	0.0335 g/L/h	Wang et al. (2021)
	*Kluyveromyces marxianus* C21	4.0	35	60	Okara, MSG, peptone	4.31 g/L	0.072 g/L/h	Zhang et al. (2022)

#### Two-stage fermentation

Within the field of industrial biotechnology, the two-stage fermentation process has demonstrated exceptional efficacy in the synthesis of GABA, a bioactive molecule that offers notable health advantages. This approach uses the complicated metabolic control of microorganisms, by modifying culture conditions and nutrition availability, to optimize GABA production. The investigation of *Lactobacillus brevis* CGMCC 1306, which exhibited its greatest proliferation at 35 °C and 5 pH, serves as an illustrative example. On the contrary, the optimal conditions for GABA synthesis were slightly different: a higher temperature of 40 °C and a slightly more acidic pH of 4.5 (Chunlong et al. 2013). As a consequence of this differentiation in reaction pathology between the growth and GABA production stages, a two-stage fermentation methodology is necessary. During the initial 32 h of the first stage, optimal conditions for cell growth are maintained at 35 °C and pH 5.0. Subsequently, the conditions are adjusted to 40 °C and pH 4.5, factors that stimulate the synthesis of GABA. Through the implementation of this strategic modification, the concentration of GABA increased substantially within 72 h to 475 mmol L^−1^, representing a remarkable achievement in comparison to the 399 mmol L^−1^ generated in single-stage fermentation systems. Following the initial concentration of the substrate being adjusted to inhibit cellular proliferation, a further inoculation of the substrate was executed after a duration of 56 h; this action led to a GABA yield increase to 526 mmol L^−1^ (Pannerchelvan et al. 2023).

An additional inquiry was conducted to assess the effectiveness of a two-stage fermentation procedure that combined roasted soybean flour (TRSF) and turmeric, utilizing *Bacillus subtilis* HA and *Lactobacillus plantarum* K154. According to the findings of Lim et al. (2016), the efficacy of the method improved by 1.78% in comparison to a single-stage fermentation approach. A comparable 1.47% increase in GABA production was observed when *Bacillus subtilis* HA and *Lactobacillus plantarum* EJ2014 were added to Cucurbita mascara during fermentation (Park et al. 2019). The methodology entailed the inoculation of *B. subtilis* with 5% MSG for an initial period of one day. Following this, *L. plantarum* EJ2014 was introduced into the mélange, which was then incubated for an extra 7 days.

Metabolism must be strictly regulated in order for these processes to function. After achieving the ideal levels of GAD production, it is possible to enhance the GABA yield by optimizing the fermentation parameters to take advantage of the biotransformation capabilities exhibited by the LAB cells. The initial temperature for *Streptococcus salivarius* subsp. thermophilus Y2 was determined to be 37 °C in order to achieve optimal GAD production. Subsequently, the pH was adjusted to 4.5 and the temperature was increased to 40 °C in anticipation of the GAD reaction. The introduction of 0.02 mmol L^−1^ PLP at this juncture resulted in a 1.76-fold increase in the yield of GABA generated, which surpassed the output of a single-stage fermentation process (Yang et al. 2008). The adeptness and flexibility with which this methodology regarding fermentation conditions was implemented underscore the intricate nature of microbial metabolic regulation in the effective production of GABA.

#### Co-culture fermentation

Co-culture fermentation has become a crucial approach in the field of fermentation methods utilized for the commercial production of GABA, especially when applied to two-stage fermentation processes. Typically, this methodology entails the collaborative operation of a collection of microorganisms, frequently belonging to the same family or possessing metabolic characteristics that complement one another. As an illustration, within this particular framework, precursors generated by one microorganism may be employed by another to synthesize the intended product, GABA (Cui et al. 2020). The metabolic regulation of each organism in these co-culture systems is dynamically responsive to nutrient availability and culture conditions. The interaction described has the potential to greatly improve the effectiveness and output of the fermentation procedure. As an illustration, Watanabe et al. (2011) reported that the co-cultivation of *S. thermophilus* IFO13957 and *Lb. delbrueckii* subsp. bulgaricus IAM1120 resulted in a noteworthy augmentation in the synthesis of GABA, which peaked at 15 mM. In contrast, the individual cultures of these microorganisms yielded considerably lower concentrations of 0.25 mM and 0.15 mM, respectively. *L. brevis* 877G, an additional strain that is frequently employed in co-culture fermentation, demonstrates diminished proteolytic activity when used alone in milk fermentation (Wu et al. 2015). This is attributed to the lack of extracellular proteinase encoding genes in *L. brevis* 877G. On the contrary, a synergistic effect is observed when conventional dairy starters, which demonstrate enhanced proteolytic activity and are capable of degrading milk protein into peptides and additional nutrients, are co-cultured with the former. The nutrient-dense environment created by this interaction promotes the proliferation of *L. brevis* cells, consequently augmenting the fermentation process (Cui et al. 2020). One prominent example of this synergy can be found in the co-cultivation of *L. sakei* 795 and *L. brevis* 877G, which resulted in a noteworthy increase in GABA concentration to 22.51 mM during the process of milk fermentation (Seo et al. 2013b). Furthermore, Karimian et al. (2020) reported that the co-cultivation of *L. plantarum* and *L. lactis* subspecies lactis, which was obtained from cheese whey, resulted in a peak GABA production of 366 mg per 100 mL. In a subsequent study, researchers examined the effectiveness of combining *Lactobacillus plantarum* K154, isolated from kimchi, and *Leuconostoc mesenteroides* SM, derived from carrot juice, to ferment *Oenanthe javanica* DC, also known as water dropwort, and produce a functional food rich in GABA. The study revealed that the acidic environment created during the early stages of fermentation by *Leu. mesenteroides* SM substantially boosted the GABA production capacity of *L. plantarum* K154. As a result, the GABA concentration significantly increased from 1.5 mg/mL, achieved with *L. plantarum* K154 alone, to 100 mM in the co-culture fermentation setup (Kwon et al. 2016). In a two-step fermentation process, co-culturing LAB *L. futsaii* CS3 and the fungus Candida rugosa 8YB generated the greatest amount of GABA (135 mg mL^−1^ h^−1^) (Sanchart et al. 2018). The procedure entailed the preliminary synthesis of l-glutamic acid by *L. futsaii* CS3, which was subsequently employed in the subsequent fermentation phase to produce GABA. In addition, an economically viable co-culture of *L. plantarum* K154 and the fungus *Ceriporia lacerate* yielded active peptides and polysaccharides in addition to a substantial quantity of GABA (15.53 mg mL^−1^) (Lee and Lee 2014). Therefore, the efficacy of co-culture fermentation in the synthesis of GABA is dependent on the precise metabolic regulation of the microorganisms involved, which is adjusted meticulously in accordance with particular culture conditions and the availability of nutrients. The increased efficiency and productivity observed in these fermentation systems are predicated on this metabolic interaction between co-cultured strains.

### Modifying metabolic pathway of GABA

#### Regulation of GAD activity

A key tactic for boosting GABA production and improving GABA bioconversion is targeted metabolic pathway regulation via genetic engineering. Utilizing genetic engineering as a way to enhance GAD activity is a beneficial approach (Choi et al. 2015). The direct modulation approach and indirect modulation approach are the two different pathways to improve GABA production via changes in the metabolic pathways of bacteria. In the direct modulation approach, the decarboxylation activity of l-glutamic acid into GABA has been enhanced by the GAD-encoding gene. In the meanwhile, the alteration of cell metabolism indirectly affects cell growth and improves GABA production by indirect modulation approach (Pannerchelvan et al. 2023). The process engineering approach would effectively deduct the overall production cost along with no chemical residue and high yield with an ideal way to produce value-added compounds (Yuan and Alper 2019). Hence different fermentation modes, process optimization and control, whole-cell bioconversion, physiological-oriented strategy, and new co-culture systems are some of the advanced approaches experts have looked forward to (Luo et al. 2021).

The overexpression of key genes encoding GAD is a distinctive metabolic engineering direction to GABA synthesis by LAB. According to Tajabadi et al., overexpressing the gad gene with the pMG36e vector in *L. planatarum* yielded a higher GAD activity and produced 1.14 g/L GABA which is greater than 55% that of the wild type strain (Yuan et al. 2020). Therefore, overexpressing key enzymes for the construction of a gene expression system is the significant pathway to achieve a high titer of GABA. Based on this principle, engineered *L. brevis* pMG36e-gadA has generated a higher cell-bound activity for GAD as compared to other strains while cloning gadA, gadB, gadC, gadCB, and gadCA (Lyu et al. 2017). Moreover, Lyu et al. (2017) and Jaichumjai et al. (2010) have concluded that GAD activity can also be achieved by reducing the activity of FF-ATPase. An FF-ATPase deficient strain NRA6 isolated from *L. brevis* pMG36e-gadA from the bromocresol green (BCG) media produced 43.65 g/L of GABA with the 98.4% of the conversion rate of MSG while control strain *L. brevis* pMG36e only produced 35.81 g/L of GABA under the similar conditions. Other than that, Lyu et al. (2018) found an interesting fact deletion of mutations in the gad genes of *L. brevis* CGMCC1306 has affected acid tolerance ability and GAD activity. According to the study of Lyu et al., *L. brevis* 9530: pNZ148-gadBC produced 1.45 g/L/h of GABA with higher GAD activity of 104.34 g/L. A recent study in 2020, has found that, the expression of gadB1, and gadC1 in *lactococcus lactis* F44 produced 9.12 g/L of GABA but co-expression of *Lactococcus lactis* CV56 on *Lactococcus lactis* NZ9000 produced a higher GABA titer of 25.61 g/L with a productivity of 0.711 g/L/h (Liu et al. 2020; Lyu et al. 2020).

By encoding the ncgl0464 to *C. glutamicum* GABA specific transporter (GabPcg), a GRAS bacterium, *C. glutamicum* has played a significant role in the absorption and production of GABA (Ruan et al. 2020). A medium without the addition of MSG of l-glutamate with *C. glutamicum* ATCC 13032 has yielded 2.15 g/L of GABA from 160 g/L of glucose with productivity of 0.030 g/L/h by expressing pDXW-8/gadRCB2 genes encoding GAD, GadC and transcriptional regulator. Although the GABA titer was relatively low it excluded the acquisition of precursor (Shi and Li 2011). However, with the aim of enhancing GABA production by glucose medium, two GAD genes of gadB1 and gadB2 isolated from *L. brevis* LB85 were co-expressed in *C. glutamicum* have indicated that 27.13 g/L in flask-based batch fermentation (Shi et al. 2013). The co-factor PLP has directed for the common GAD systems in the GABA synthesis pathway. Even though the high cost of PLP, pyridoxal kinase (PLK) encoded by the plk gene catalyzes the ATP-dependent phosphorylation reaction of pyridoxal to produce PLP. Based on this principle *Lb. planatarum* GB 01–21 co-expressed with *C. glutamicum* yielded a better GABA performance of 70.6 g/L of titer along with 1.01 g/h/L productivity without MSG medium (Zhang et al. 2014b).

#### Engineered MSG pathway

In recombinant *E. coli*, three genes (gadA, gadB, and gadC) encoding GadA, GadB, and GadC were cloned and individually or collectively ligated into the plasmid pET32a to create expression plasmids. These expression plasmids were then transformed into *E. coli* BL21(DE3) to generate strains capable of biosynthesizing GABA. Upon induction, these strains produced GABA by converting l-glutamate through glutamate decarboxylase activity and the glutamate/GABA antiporter system (Somasundaram et al. 2017; Yu et al. 2018). According to the investigation of Yu et al., *E. coli BL21(DE3)*/pET32a-gadA, *E. coli BL21(DE3)*/pET32a-gadAB, and *E. coli BL21(DE3)*/pET32a-gadABC—were engineered to enhance GABA production using MSG. It has been resulted that, the highest GABA titer was obtained from the *E. coli BL21(DE3)*/pET32a-gadABC which was 3.98 g/L from 10 g/L of MSG. It was a relatively higher value compared with GadA and GadAB which were 1.25 g/L and 2.31 g/L respectively from 10 g/L of MSG. The investigation concluded that GadC can overexpress to shift glutamate into the cell and pump GABA out (Yu et al. 2018). A similar study which was done in 2012 found that 5.46 g/L of GABA was yielded from 10 g/L of MSG from GadB when *E. coli* XL1 was overexpressed in *E. coli* XB (Le Vo et al. 2012).

### Whole-cell and vitro conversion of GABA

Whole-cell conversion uses genetically modified organisms, like *E. coli* or yeast, engineered to overexpress GABA-producing enzymes. These enzymes convert substrates like glutamate or glucose into GABA through fermentation, offering a scalable, cost-effective method with minimal equipment needs (Ke et al. 2016). In this study, the authors employed *Escherichia coli*’s Glutamate Decarboxylase B (GadB) for the synthesis of GABA. To achieve efficient GABA production, the researchers cloned and overexpressed GadB in *E. coli* using a high copy number plasmid. A key aspect of the method involved subjecting the whole cells to a 24-h cold treatment at − 20 °C, which prompted GadB to migrate to the periplasm. This strategic approach led to a notable enhancement in the enzymatic turnover rate, resulting in a two-fold increase in GABA production. The results were impressive, with a 100% conversion of MSG to GABA achieved through this methodology. Initially, the production rate was established at 46.9 g/L GABA, but further optimization efforts led to an unprecedented yield of 850 g/L GABA. These findings demonstrated the efficiency and scalability of the developed method. Notably, the whole-cell biocatalysts could be recycled up to ten times, contributing to the process’s sustainability (Xue et al. 2021).

Advances in whole-cell conversion, such as using Lactobacillus brevis and engineered *E. coli*, have led to significant increases in GABA production. *Lb. brevis* TCCC 13007, under pH-controlled conditions, achieved a GABA accumulation of 38 g/L, while engineered E. coli reached a record-breaking production of 308.96 g/L with 99.9% conversion in 12 h, yielding a total of 614.15 g/L. These methods highlight the potential of whole-cell bioconversion as a cost-effective industrial resource (Zhang et al. 2012). Another study developed a simple and highly efficient way for the synthesis of GABA by using engineered *E. coli* as a whole-cell biocatalyst from l-glutamic acid (l-Glu). The highest production of GABA reached 308.96 g L^−1^ with 99.9 mol% conversion within 12 h, when *E. coli* Δ gabAB (pRB-lgadB) concentrated to an OD 600 of 15 in 3 M l-Glu at 45 °C. The total GABA yield reached 614.15 g L^−1^ with a molar yield over 99%, which represented the highest GABA production ever reported (Ke et al. 2016). Therefore, the whole-cell bioconversion system allowed us to achieve a promising cost-effective resource for GABA in industrial application.

The conversion of GABA in vitro conversion typically involves enzymatic or chemical methods to modify GABA’s structure, creating different compounds. A common process is the conversion of GABA into succinic semialdehyde via transamination, catalyzed by GABA transaminase (Parviz et al. 2014). The process of metabolizing GABA in a controlled environment is intricate and affected by several factors, such as the quantity and origin of l-glutamate, which acts as the precursor for GABA. The presence of glutamate is crucial in controlling the synthesis of GABA, since increased levels stimulate its production, while excessive quantities can impede bacterial development and decrease GABA yields. It is crucial to optimize the pH and temperature conditions for the generation of GABA by different bacterial strains. The appropriate pH range is around 5.0, and the optimal temperature is 37 °C (Rayavarapu et al. 2021). Furthermore, the proper functioning of glutamic acid decarboxylases (GADs), which are enzymes responsible for synthesizing GABA, is dependent on the presence of essential cofactors such as pyridoxal 5′-phosphate (PLP) and other nutrients. On the other hand, the existence of certain inhibitors, such as some medications, can have an adverse effect on GABA metabolism (Jewett and Sharma 2018).

These factors determine the efficiency and yield of GABA production. For instance, one study reported that the highest GABA production (6.03 g/L) was achieved by using a bacterial cellulose membrane-immobilized GAD from *Lactobacillus brevis* at pH 5.4–5.6, 45 °C, and 0.1 M glutamate (Yao et al. 2013). Another study focuses on the in vitro production of GABA using seven probiotic strains and MSG-supplemented medium. Key findings include *Levilacto bacillus brevis* LB01 and *Lactiplanti bacillus* plantarum 299v being the most effective GABA producers. The research utilized anaerobic faecal batch cultures with gut model medium, also MSG-enriched, incubated at 37 °C and pH 5.4–5.6. It tested GABA production under six different scenarios, including the use of prebiotic OFI, over 48 h, to simulate the human proximal colon’s conditions for GABA study (Monteagudo-Mera et al. 2023).

The result highlights how substantial improvements in GABA production in vitro can be achieved by optimizing reaction conditions; it complements the developments observed in whole-cell conversion techniques and the wider domain of GABA research. This method allows for controlled reactions and high yields of pure products but may require expensive starting materials and may not be scalable. While both methods have their advantages, whole-cell conversion appears more promising for large-scale industrial applications due to its cost-effectiveness and scalability. However, in vitro methods remain valuable for specific applications where precise control over reaction conditions is required.

## Determination of Glu and GABA

A simple and dedicated quantitative method to determine GABA is very important for the biotechnology industry. Diverse methods have been utilized to detect the levels of GABA and Glu, including thin-layer chromatography, ion-exchange separation coupled with post-column derivatization, high-performance liquid chromatography (HPLC) with fluorescence detection, HPLC with UV detection, gas chromatography–mass spectrometry (GC–MS), capillary electrophoresis, and liquid chromatography–mass spectrometry (LC–MS) (Cao et al. 2013; Defaix et al. 2018; Ji et al. 2023; Kehr 1998; Suñol et al. 1988). However, among these diverse techniques, spectrophotometric and HPLC-based methods are widely manipulated to determine the GABA content. A chemically modified derivative applied to GABA has shown significant absorption as GABA has a weak absorption at the UV and visible range and fluorescence spectrum too (Li et al. 2019). The color intensity of spectrophotometric methods is impacted by pH, the temperature of the medium, the cooling process, and the presence of some amines, and the optical density can be read at 630 nm (Sarak et al. 2020). As mentioned previously, to enhance better absorption in the UV, visible, and fluorescence spectra, several GABA derivatives such as dansyl chloride, (Fang et al. 2020) *o*-phthalaldehyde (OPA), (Oh et al. 2019) phenylisothiocyanate and 6-aminoquinolyl-*N*-hydroxy succinimidyl carbamate have been employed (Zhou et al. 2019). Among them, derivatization with OPA can decrease the polarity and increase the retention in reversed-phase chromatography resulting in higher sensitivity to absorb UV, visible, and fluorescence. However, it is less stable and mandated to control the time of the reaction and injection (Mengerink et al. 2002; Steed 2010). A study by Farthing et al. (2017) quantified both GA and GABA in brain tissue by GC–MS/MS as a rapid and selective method for detection and quantification. In this method, they were used to stable isotopes of each compound along with MethElute™ reagent which was quickly derivatized GA and GABA and their isotopologues in the heated GC injection port. Employing low thermal technology (LTM) it assumed only 20 s to finalize and furnish high-resolution fast chromatography. This novel method detected excellent linearity from 0.5 to 100 µg/mL with the limits of detections of 100 ng/mL and 250 ng/mL for GA and GABA respectively.

Though HPLC, GC–MS, LC–MS, etc. have some drawbacks such as the degradation of derivatives, formation of numerous derivatives, high cost, and longer retention times that depleted a larger volume of solvents (Ngernsutivorakul et al. 2018). Thus electrochemical procedures have been employed widely as a result of rapid, non-destructive, powerful, sensitive, and accurate analytical assays for detecting very low concentrations of biomarkers, and environmental and organic threats without any pretreatments (El-Said and Choi 2019; El-Said et al. 2015). Recently enzyme-based or non-enzymatic electrochemical biosensors grabbed more attention for GABA determination. Glutamate oxidase (GluOx) or dehydrogenase (GLDH) enzymes have demonstrated high sensitivity though they have lower durability and high cost. Moreover, non-enzymatic electrochemical biosensors are formulating higher sensitivity and selectivity compared with enzymatic biosensors (Schultz et al. 2020).

## Market analysis and insights

The GABA market is a captivating exploration of the human body and mind, with implications for mental well-being and physiological balance. The increased prevalence of neurological disorders, as well as consumer demand for natural and organic dietary supplements, are driving the global GABA market. Furthermore, GABA can be classified according to its manufacturing method, intended application, and region in the worldwide market. The market is divided into two categories based on type of production: chemical synthesis and biological fermentation. Chemical synthesis involves a more complex reaction process than does biological fermentation, making the former far more promising. It also has a mild response, a high catalytic efficiency, and is ecologically friendly (MarketWatch 2023). The GABA market is expanding as a result of rising health awareness, holistic wellness, mental health awareness, confirmation of scientific research, new products, customer experience, shift toward self-care, and improved information availability. With its adaptable and scientifically proven advantages, GABA is useful to the food and beverage industry, chemical industry, and cosmetics industry. As an example GABAergic medicines including in GABA pills are used for emotional distress and sleep (Research 2022). In the United state of America (USA), GABA is a frequently used ingredient in nutritional supplements for treating anxiety, mood disorders, premenstrual syndrome (PMS), increasing lean muscle mass, burning fat, regulating blood pressure, and treating pain (Yahoo!finance 2023). Moreover, it has demonstrated potential as an animal feed addition by increasing feed intake and animal weight (Dataintelo 2022). Its natural beginnings have surpassed its biological origins, and it has become a symbol of tranquility in a challenging global landscape. According to geography, the market is divided into North America, Europe, Asia Pacific, Latin America, and Middle East and Africa (MEA) (Dataintelo 2022). The GABA Supplements Market is anticipated to reach $76 million by the end of 2033 and expand at a CAGR of 5.7% between 2023 and 2033. Besides, to the following year, it is anticipated that the U.S. would control more than half of the market because of GABA’s broad use in the US market, U.S. Pharmacopeia (USP) created a dietary supplement quality monograph. Following USP requirements for dietary ingredient admittance, the monograph included a safety review (USP 2020; Yahoo!finance 2023). Some GABA manufacturers and information are summarized in Additional file 1.

## Conclusions and future prospects

GABA is a vital neurotransmitter found in the brain and central nervous system of animals and present in a variety of other living organisms. Its multifaceted utility, which includes its function as a sedative, therapeutic properties in treating epilepsy, reducing cancer cell proliferation, and extensive use in pharmaceuticals and functional foods, highlights its importance. With the growing demand for GABA, particularly for mass production in the food and pharmaceutical industries, its commercial value has significantly increased. The biosynthetic approach, specifically microbial fermentation, has emerged as the most effective method for GABA production, primarily due to its safety, environmental sustainability, and high yield. Novel techniques are continually being developed and optimized to meet the increasing demand for GABA in the food and pharmaceutical industry. Despite significant progress in GABA research, several knowledge gaps remain, including an incomplete understanding of its biosynthetic pathways in non-model organisms and under variable environmental conditions, and a need for cost-effective and sustainable biotechnological strategies for large-scale production. Furthermore, there is a need to optimize GABA delivery systems for improved bioavailability and targeted therapeutic outcomes, investigate its potential in mitigating metabolic disorders, and examine its effects on immune modulation through extensive clinical trials. Integration of advanced technologies and robust experimental methodologies are essential to address these knowledge gaps and fully exploit GABA’s multifunctional properties, ultimately leading to transformative discoveries and practical applications with significant societal impact. Recent advances in genetic and metabolic engineering have led to significant breakthroughs in GABA production. Overexpression of the GAD gene in lactic acid bacteria, such as *Lactobacillus plantarum*, has been shown to increase GABA production. Additionally, metabolic engineering techniques, such as using the pMG36e-gadA construct in *Lactobacillus brevis*, have resulted in elevated cell-bound GAD activity. Furthermore, reducing FF-ATPase activity in certain strains has led to higher GABA yields. In an effort to move towards more sustainable and eco-friendly practices, renewable resources like lignocellulosic biomass, glucose, and glycerol are being used for microbial GABA production in lactic acid bacteria strains. Genetic modification of *Escherichia coli* strains has also contributed to advancements in GABA biosynthesis, including disruptions in the tricarboxylic acid (TCA) and glyoxylate cycles and modifications to the GABA production pathway, resulting in substantial improvements in GABA output. To address pH inconsistencies in *Corynebacterium glutamicum*, researchers have expressed mutant GADs from various sources, leading to improved pH stability and increased GABA production. Further genetic modifications in this organism, such as encoding GABA-specific transporters and expressing genes like pDXW-8/gadRCB2, have demonstrated impressive efficacy in batch fermentation. These cutting-edge methodologies hold great potential for transforming GABA manufacturing on a large scale while minimizing environmental impact. Continued research and refinement of these cutting-edge methods will likely open up new avenues for the large-scale production of GABA. Further investigation is needed to uncover additional roles for GABA in both human and microbial systems, with a focus on ensuring the molecular safety of the relevant strains. However, optimizing and scaling up production processes is crucial to achieve remarkable quantities of this valuable compound.

### Supplementary Information

**Additional file 1.** GABA manufacturers and information.

## Data Availability

Data sharing not applicable to this article as no data sets were generated or analyzed during the current study.

## Abbreviations

ODHC: 2-Oxoglutarate dehydrogenase
*C. glutamicum*: *Corynebacterium glutamicum*
CP: C peptide
GLDH: Dehydrogenase
GABA: Gamma-aminobutyric acid
GABA-AT: Gamma-aminobutyric acid aminotransferase
GABA-T: GABA transaminase
GC–MS: Gas chromatography/mass spectrometry
GRAS: Generally recognized as safe
GSMM: Genome-scale metabolic model
GDH: Glutamate dehydrogenase
GluOx: Glutamate oxidase
GAD: Glutamic acid decarboxylase
HPLC: High-performance liquid chromatography
IRG: Immunoreactive glucagon
IRI: Immunoreactive insulin
LAB: Lactic acid bacteria
LC–MS: Liquid chromatography/mass spectrometry
LTM: Low thermal technology
MEA: Middle East and Africa
OCD: Obsessive–compulsive disorder
OPA: *O*-Phthalaldehyde
PEPC: Phosphoenolpyruvate-carboxylase
PTSD: Post-traumatic stress disorder
PMS: Premenstrual syndrome
PTKs: Protein kinase
PLP: Pyridoxal-5′-phosphate
TRSF: Roasted soybean flour
SSADH: Semialdehyde dehydrogenase
SA: Succinic acid
SSA: Succinic semialdehyde
TCA: Tricarboxylic acid
USP: U.S. Pharmacopeia
USA: United States of America
