# Supplementary material for: Recent advances in the biosynthesis and industrial biotechnology of Gamma-amino butyric acid
Source: Bioresour Bioprocess. 2024 Mar 16;11(1):32. doi: 10.1186/s40643-024-00747-7 (PMC10992975; doi:10.1186/s40643-024-00747-7)
Supplement: Supplementary file 1 — Additional file 1. GABA manufacturers and information. [file 40643_2024_747_MOESM1_ESM.docx]

**Additional file 1.** GABA manufacturers and information

| Company/Country | Method | Product trademark | website |
| --- | --- | --- | --- |
| Sky premium life/Greece | Chemical synthesis | GABA | www.skypremiumlife.com |
| BIOMENTA/ Germany | Chemical synthesis | Nacht Sterne | www.biomenta.de |
| USDA Organic/Japan | Fermentation | GABA OOLONG- CERTFIEDORGANIC | yunnansourcing.us |
| Thorne Research/USA | Fermentation | PharmaGABA-100 | www.thorne.com |
| Vitaminesperpost.nl/Netherlands | Chemical synthesis | GABA | www.vitaminesperpost.nl |
| Bloomage Biotech/China | Fermentation | GabacareTMBL98T | www.bloomagebioactive.com |
